# 

**Published:** 1906-09-22

**Authors:** 


					ihs *osffiM^^ (1;. n,;l iiuii im
PITA Lr
\r^ i ^
uAscory Western Infirmary
1.044. Vol. XL rK?fiatered as~| Saturday,
? |_a Newspaper. J
Sept. 22nd, 1906.
[3nbsereKnoTerms] PRICE THREEPENCE

				

